# Predictors of right ventricular function improvement with sacubitril/valsartan in a real‐life population of patients with chronic heart failure

**DOI:** 10.1111/cpf.12726

**Published:** 2021-09-28

**Authors:** Michele Correale, Pietro Mazzeo, Michele Magnesa, Martino Fortunato, Lucia Tricarico, Alessandra Leopizzi, Adriana Mallardi, Raffaele Mennella, Salvatore Tucci, Natale Daniele Brunetti

**Affiliations:** ^1^ Ospedali Riuniti University Hospital Foggia Italy; ^2^ Department of Medical and Surgical Sciences University of Foggia Foggia Italy

**Keywords:** ARNI, chronic heart failure, neprilysin inhibitors, right ventricular function, sacubitril valsartan

## Abstract

**Background:**

Observational studies have demonstrated that treatment with sacubitril/valsartan may improve left ventricular (LV) systolic and diastolic function in subjects with reduced LV ejection fraction (LVEF) in real‐world studies. Subjects with heart failure and reduced EF (HFrEF), however, are also characterized by an impaired right ventricular (RV) function. We therefore aimed to evaluate whether also RV function may improve after S/V therapy and possible predictors of RV improvement could be identified at echocardiography and tissue Doppler imaging.

**Methods:**

Fifty consecutive patients (67 ± 8 years, LVEF 28 ± 6%, male 86%) with chronic HFrEF and NYHA class II‐III were followed up for 6 months after therapy with S/V. LV&RV function was assessed at baseline and after 6 months of therapy.

**Results:**

After 6‐month therapy with S/V a significant improvement was shown in the following echocardiography parameters assessing RV function: PAsP (31 ± 11 vs. 35 ± 10 mmHg, *p* < 0.001), TAPSE (19 ± 3 vs. 18 ± 3 mm, *p* < 0.001), RV FAC (38 ± 7 vs. 34 ± 6 mm, *p* < 0.001), RV S’ (12 ± 2 vs. 10 ± 2 cm/s, *p* < 0.001), RV‐FW‐LS (−20 ± 5 vs. −18 ± 5%, *p* < 0.001), RV‐4Ch‐LS (−16 ± 5 vs. −14 ± 5%, *p* < 0.001). At multivariable analysis improvement in RV‐FW‐LS was associated to baseline levels of RV S’ (*r* 0.75, *p* < 0.01) and RAV (*r* –0.32, *p* < 0.05).

**Conclusions:**

In a real‐world scenario, 6‐month therapy with S/V was associated with an improved RV function in HFrEF. RV function improvement may be predicted by assessing baseline RV S’ and right atrial volume values.

## 1 BACKGROUND

The use of angiotensin receptor/neprilysin inhibitors (ARNI) is currently recommended for the treatment of heart failure with reduced ejection fraction (HFrEF); in the PARADIGM‐HF trial (McMurray et al., 2014), treatment with sacubitril/valsartan (S/V) was associated with a 20% reduction in the primary composite endpoint of cardiovascular death or HF hospitalization compared with enalapril.

Previous studies have demonstrated that S/V treatment may improve left ventricular (LV) systolic and diastolic function (Martens et al., 2018) and reverse remodelling (Almufleh et al., 2017) in subjects with HFrEF. S/V also reduced costs of hospitalization in real‐world registries (Correale et al., 2019). Recently, clinical parameters have been shown to predict the response to S/V therapy in a real‐world scenario of patients with chronic HF (CHF) (Correale et al., 2021).

Subjects with HFrEF, however, may also present an impaired right ventricular (RV) function. The presence of RV dysfunction indicates HF progression and may carry an additional worse prognostic value (Carluccio et al., 2018). RV recovery is associated with an improved outcome (Dini et al., 2016), so the assessment of RV function has been increasingly studied, also with echocardiography (Mercurio et al., 2018). Peak longitudinal strain of RV free wall (RV‐FW‐LS) (not including the interventricular septum), an angle‐independent and less load‐dependent parameter, has been recently proposed as a more accurate and sensitive tool to evaluate RV function. Measurement of RV longitudinal deformation that includes the analysis of the interventricular septum in the region of interest is called RV four‐chamber strain (RV‐4Ch‐LS). In patients with HFrEF, RV global longitudinal strain showed incremental prognostic value over left ventricular ejection fraction (LVEF) (Motoki et al., 2014). Carluccio et al. demonstrated that in patients with HFrEF and preserved TAPSE (tricuspid annular plane systolic excursion), RV‐FW‐LS provides incremental prognostic information and improves risk stratification (Carluccio et al., 2018).

On these bases, we therefore sough to assess, in a real‐world scenario of patients with HFrEF, whether therapy with S/V may improve RV function, also assessed by using tissue Doppler imaging modalities (speckle‐tracking strain), and whether possible predictors of RV improvement could be identified.

## 2 METHODS

Fifty consecutive patients with HFrEF in NYHA functional class II‐III, enrolled in the Daunia Heart Failure Registry as reported elsewhere (Correale et al., 2011, 2012, 2015), were followed up between September 2019 and March 2020. Enrolment criteria included LVEF ≤35%, systolic blood pressure ≥100 mmHg, eGFR ≥30 ml/min/1.73 m², potassium levels ≤5.4 mmol/L. All patients were treated with stable ACE‐inhibitor or angiotensin receptor antagonist doses for at least 6 months and started treatment with sacubitril/valsartan therapy as recommended by the 2016 ESC guidelines on HF diagnosis and treatment (Ponikowski et al., 2016). Medical history, heart rate, systolic blood pressure, body mass index, NYHA functional class and medications were recorded and monitored. All patients underwent blood analysis, ECG, conventional and advanced echocardiography with TDI and speckle tracking for assessment RV function in an ambulatory setting under resting conditions at the beginning and after 6 months of therapy with S/V.

### 2.1 Echocardiography

Conventional echocardiography was used to assess LV dimensions and LVEF, peak velocities of trans‐mitral early (E) and late diastolic (A) LV filling, the ratio of trans‐mitral early to late (E/A ratio) LV filling velocity. LV dimensions and LVEF were calculated as recommendations in the joint ASE/ESC guidelines (Lang et al., 2015). LVEF was calculated according to the biplane method of discs (modified Simpson method). It is a 2D echocardiographic technique requiring area tracings of the LV cavity. It requires tracing the LV endocardial border in the apical 4‐ and 2‐chamber views in both end‐diastole and end‐systole. The tracings are used to divide the LV cavity into a predetermined number of discs (usually 20) with disc volumes based on the tracings.

The left atrium volume index (LAVi) was obtained by tracing along the endocardium of the LA in both relaxation and contraction phases, starting at the annulus, passing through the roof and ending back at the annulus in a four‐chamber view; these measurements were made according to the current accepted standard for 2D‐TTE LA volume measurement (Lang et al., 2015), using the biplane area‐length or modified disc method.

For RV quantification, RV fractional area change (FAC%) was obtained by making a trace along the endocardium of the RV in both relaxation and contraction phases starting at the annulus, passing through apex and ending back at the annulus in a 4‐chamber view. FAC was calculated as (End‐Diastolic Area–End‐Systolic Area) × 100/(End‐Diastolic Area). Echocardiographic RA volume method included single plane Simpson's method of discs (Simpson's).

The TAPSE was measured using apical views adjusted to optimize RV structures and to achieve proper orientation for M‐mode measures.

Pulsed Doppler mitral inflow velocities were obtained by placing a 1–2 mm sample volume between the tips of the mitral leaflets in the apical four‐chamber view. The Doppler beam was aligned parallel to the flow direction. TDI measurements were recorded at the septal and lateral mitral annulus in apical four‐chamber view, included early (E’) diastolic velocities. Provision was made to average the peak lateral and septal velocities of the E’ wave. The averaged e’ was used for calculation of the E/e’ ratio (the trans‐mitral to mitral annular early diastolic velocity ratio).

RV function was assessed using tricuspid annular plane systolic excursion (TAPSE), tissue Doppler velocity of the lateral tricuspid annulus (peak systolic velocity S’), RV FAC, Pulmonary Artery systolic Pressures (PAsP) values and RV speckle‐tracking strain imaging. The TAPSE was measured using apical views, adjusted to optimize RV structures and to achieve proper orientation for M‐mode measures.

PAsP were estimated using the approach of calculating the systolic pressure gradient between right ventricle and right atrium by the maximum velocity of the tricuspid regurgitant jet, using the modified Bernoulli equation and then adding to this value the estimated right atrial pressures based on both sizes of the inferior vena cava and the change in calibre of this vessel with respiration, according to international recommendations. The peak tricuspid regurgitation velocity was assessed by placing the continuous Doppler through the tricuspid valve and the degree of tricuspid regurgitation was evaluated by the colour mode. Transthoracic echocardiography was performed using an EPIQ 7C ultrasound system (Philips Medical Systems) with X5‐1 matrix array transducer. All echocardiographic studies were performed and interpreted by experienced physicians, whom were blinded of clinical data.

### 2.2 Speckle‐tracking strain analysis for assessment right ventricular function

Speckle‐tracking strain analysis was performed for each patient with the aid of a dedicated software (Qlab 10) to measure both RV‐FW‐LS and RV‐4ch‐LS. RV focused 4‐chamber view, with the Digital Imaging and Communications in Medicine (DICOM) formatted file images, were uploaded into a personal computer for subsequent off‐line GLS analysis with AutoStrain application. Longitudinal speckle‐tracking strain was calculated applying an automated contouring detection algorithm and regions of interest manual adjustments were performed where necessary. Based on the deformation of the green endocardial contour, longitudinal strain is computed for the free wall (RV‐FW‐LS) and the global 4‐chamber contour (RV‐4Ch‐LS) and was expressed as an absolute value in accordance with current guidelines.

### 2.3 Statistical analysis

Continuous variables were expressed as mean ± standard deviation and compared with Student's *t*‐test, categorical variables as percentages and compared with χ^2^ test. Mean values were compared with Student's *t*‐test for paired groups. Linear correlations were determined by measuring the Pearson's correlation coefficient. Multivariable correction analysis was used to assess possible bias of confounders. A *p* < 0.05 was considered as statistically significant.

### 2.4 Sample sizing

On the base of prior data indicating differences in the response of matched pairs with standard deviation 3.5 mm for TAPSE (Correale et al., 2020), if the true difference in the mean response of matched pairs is 1.5 mm, we will need to study 45 pairs of subjects to be able to reject the null hypothesis that this response difference is zero with probability (power) 80%. The Type I error probability associated with this test of this null hypothesis is 0.05.

## 3 RESULTS

**FIGURE A1:**
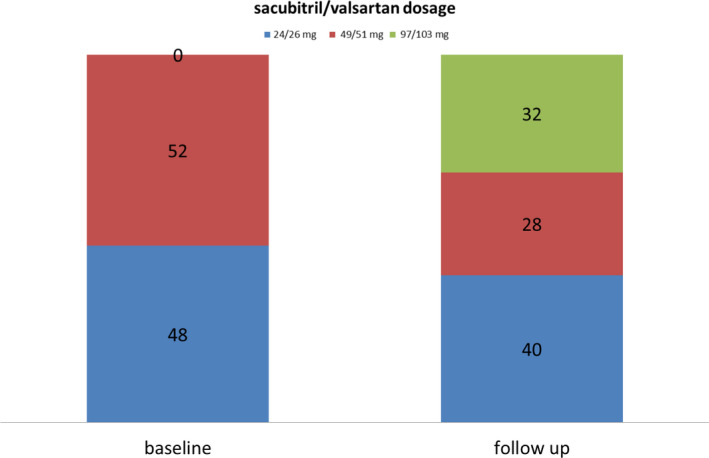
Sacubitril/valsartan dosage over time

Population characteristics (mean age 67 ± 8 years, mean LVEF 28 ± 6%, male 86%) are given in Table 1; 82% of patients were treated with beta‐blockers, 72% with ACE‐inhibitors, 28% with angiotensin receptors blockers and mineralocorticoid receptor antagonists 58%. 32% patients were on maximum dose of sacubitril/valsartan 97/103 mg b.i.d. and 28% on 49/51 mg b.i.d after 6 months of treatment (Appendix Figure A1).

**TABLE 1:** Population's characteristics

Variables	Mean ± SD	%
Age (years)	67 ± 8	
Male (%)		86%
Hypertension (%)		84%
Dyslipidemia (%)		70%
Diabetes (%)		36%
Ischaemic aetiology (%)		44%
COPD (%)		32%
LVEF (%)	28 ± 6	
ICD/CRT‐D		54%
NYHA class II (%)		62%
NYHA class III (%)		38%
SBP (mmHg)	120 ± 17	
HR (bpm)	69 ± 13	
NT‐proBNP (pg/ml)	1669 ± 3129	
Hb (gr/dl)	13 ± 1	
eGFR(MDRD)	70 ± 23	
ACE‐i (%)		72%
ARBs (%)		28%
Ivabradine (%)		19%
Mineralcorticoid receptor antagonist (%)		58%
Beta‐blocker (%)		82%

Abbreviations: ACE‐i, Angiotensin‐Converting Enzyme inhibitors; ARBs, Angiotensin Receptor Blockers; COPD, Chronic Obstructive Pulmonary Disease; CRT‐D, cardiac resynchronization therapy‐defibrillator; eGFR, estimated Glomerular Filtration Rate; Hb, haemoglobin; HR, Heart Rate; ICD, implantable cardioverter defibrillator; LVEF, Left Ventricular Ejection Fraction; SBP, Systolic Blood Pressure.

**FIGURE 1:**
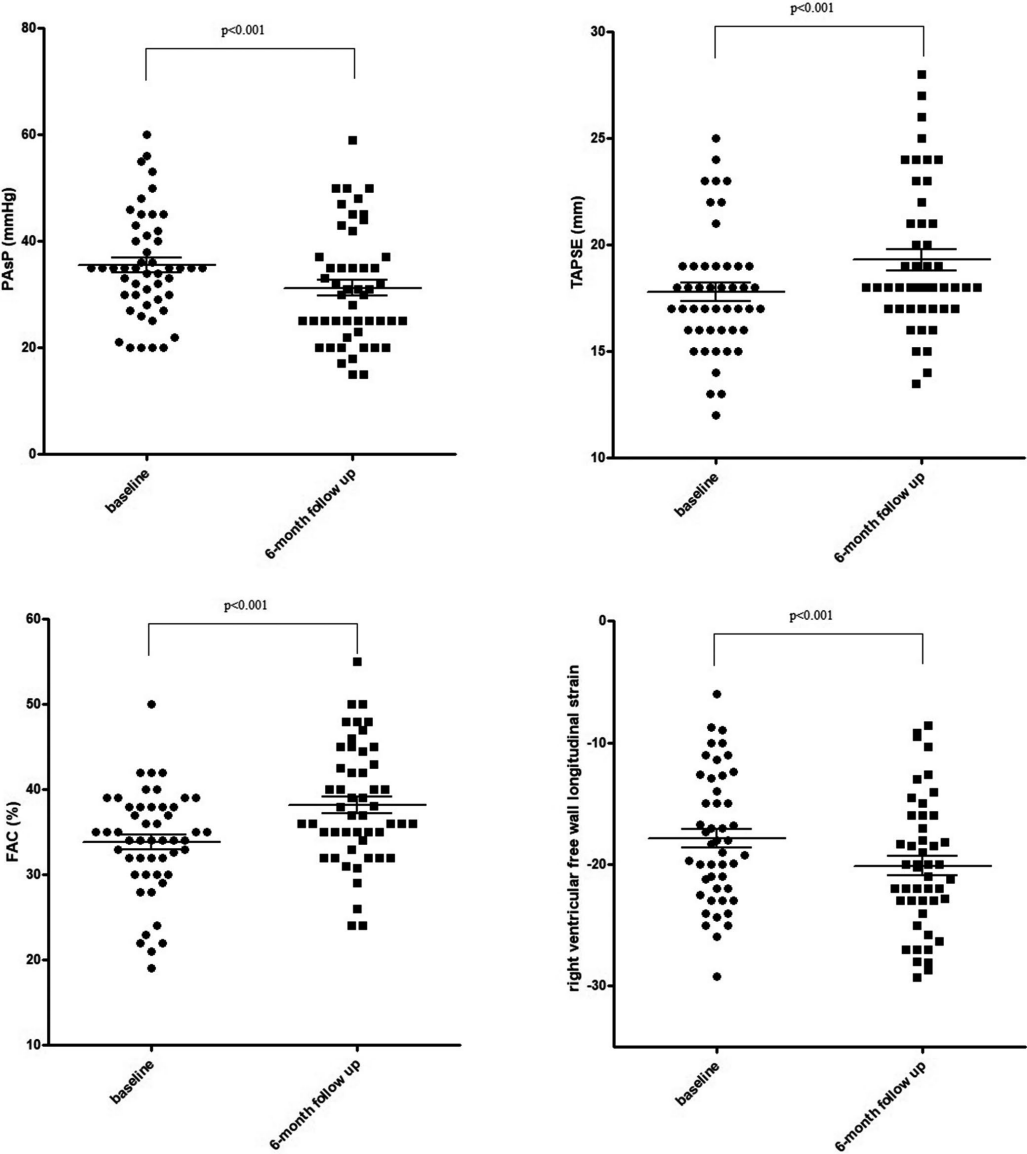
Significant changes after 6‐month therapy with sacubitril/valsartan in patients with chronic heart failure and left ventricular dysfunction

After 6 months of S/V therapy a significant improvement was shown in the following echocardiographic parameters:

1. LV function: LVEF (36 ± 7% vs. 28 ± 6%, *p* < 0.001), indexed LV end‐diastolic volume (102 ± 39 vs. 108 ± 35 ml/m^2^, *p* < 0.05), indexed LV end‐systolic volume (67 ± 29 vs. 79 ± 29 ml/m^2^, *p* < 0.001), left atrium volume index (42 ± 15 vs. 46 ± 15 ml/m^2^, *p* < 0.01),
2. RV function: PAsP (31 ± 11 vs. 35 ± 10 mmHg, *p* < 0.001), TAPSE (19 ± 3 vs. 18 ± 3 mm, *p* < 0.001), RV FAC (38 ± 7 vs. 34 ± 6 mm, *p* < 0.001), RV S’ (12 ± 2 vs. 10 ± 2 cm/s, *p* < 0.001), RV‐FW‐LS (−20 ± 5 vs. −18 ± 5%, *p* < 0.001), RV‐4Ch‐LS (−16 ± 5 vs. −14 ± 5%, *p* < 0.001) (Table 2, Figure 1).

**TABLE 2:** Comparison between baseline and follow‐up after six months of sacubitril/valsartan therapy

Parameters	Baseline (mean ±SD)	Follow up (Mean ±SD)	P‐value
LVEDD (mm)	65 ± 7	63 ± 7	<0.01
LVESD (mm)	55 ± 9	53 ± 9	<0.001
LVEDVi (ml/m^2^)	108 ± 35	102 ± 39	<0.05
LVESVi (ml/m^2^)	79 ± 29	67 ± 29	<0.001
LVEF (%)	28 ± 6	36 ± 7	<0.001
LAVi (ml/m^2^)	46 ± 15	42 ± 15	<0.010
E/E’ ratio	16 ± 6	13 ± 5	<0.001
RVD1 (mm)	38 ± 6	37 ± 7	n.s.
RVD2 (mm)	33 ± 5	34 ± 7	n.s.
RVD3 (mm)	30 ± 5	29 ± 4	<0.001
Right atrial area (cm2)	20 ± 6	19 ± 6	n.s.
RAV max (ml)	61 ± 30	60 ± 29	n.s.
TAPSE (mm)	18 ± 3	19 ± 3	<0.001
RVFAC (%)	34 ± 6	38 ± 7	<0.001
PAsP (mmHg)	35 ± 10	31 ± 11	<0.001
RV S’ (cm/s)	10 ± 2	12 ± 2	<0.001
RV−4Ch‐LS (%)	−14 ± 5	−16 ± 5	<0.001
RV‐FW‐LS (%)	−18 ± 5	−20 ± 5	<0.001

Abbreviations: E/E’ ratio, trans‐mitral to mitral annular early diastolic velocity ratio; LAVi, Left Atrial Volume index; LVEDD, Left Ventricular End‐diastolic Diameter; LVEDVi, Left Ventricular End‐Diastolic Volume index; LVEF, Left Ventricular Ejection Fraction; LVESD, Left Ventricular End‐Systolic Diameter; LVESVi, Left Ventricular End‐Systolic Volume index; PAsP, pulmonary arterial systolic pressure; RAV max, Right Atrial Volume max; RV FAC, Right Ventricular Fractional Area Change; RV FW LS, Right Ventricular Free Wall Longitudinal Strain; RV GLS 4 Ch, Right Ventricular 4‐chamber Longitudinal Strain; RV S’, right ventricular peak systolic velocity; RVD1, Right ventricular diameter 1; RVD2, Right ventricular diameter 2; RVD3, Right ventricular diameter 3; TAPSE, tricuspid annular plane systolic excursion.

**FIGURE 2:**
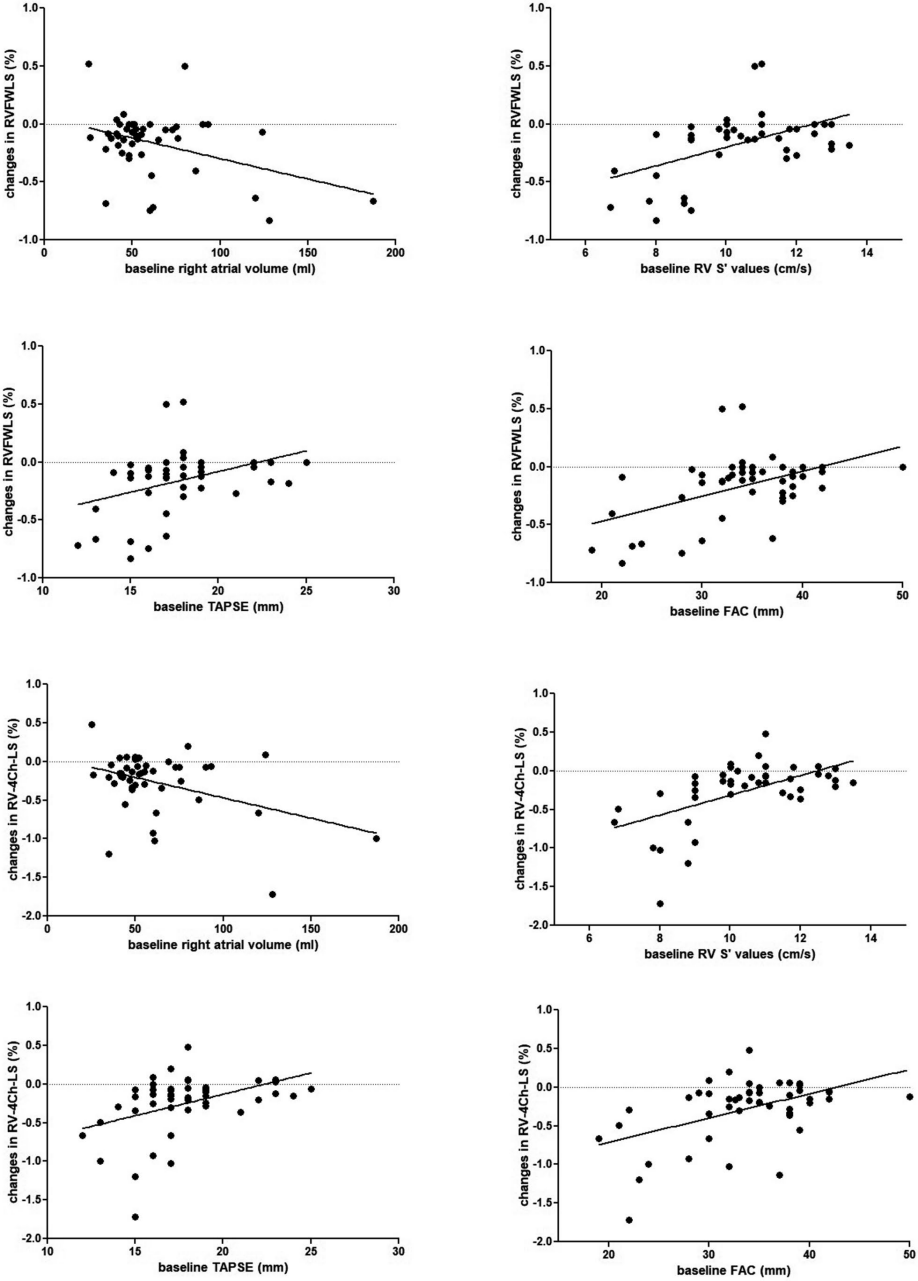
Significant correlations between baseline echocardiography parameters and changes in right ventricular longitudinal strain after 6‐month therapy with sacubitril/valsartan in patients with chronic heart failure and left ventricular dysfunction

At follow‐up, significant correlations with changes in RV‐FW‐LS were found with baseline RAV values (*r* −0.37, *p* = 0.013), baseline RAV max values (*r* −0.39, *p* = 0.008), baseline RV S’ values (*r* 0.51, *p* < 0.001), baseline TAPSE values (r 038, *p* = 0.010) and baseline RV FAC values (r 0.48, *p* = 0.001); significant correlations with changes in RV 4ChLS were found with baseline right atrial area values (*r* −0.37, *p* = 0.012), baseline RAV max values (*r* −0.40, *p* = 0.006), baseline RV S’ values (*r* 0.56, *p* < 0.001), baseline TAPSE values (*r* 0.41, *p* = 0.005) and baseline RV FAC values (*r* 0.48, *p* = 0.001) (Figure 2).

At multivariable regression analysis including age, gender, LVEF, FAC, PAsP, TAPSE, changes in LVEF and LVEDV, changes in RV‐FW‐LS were significantly associated to baseline levels of RV S’ (beta 0.75, *p* < 0.01) and RAV max (beta −0.32, *p* < 0.05), while changes in RV‐4Ch‐LS were associated to baseline levels of RV S’ (beta 0.91, *p* < 0.01) and RAV max (beta −0.28, *p* < 0.06) (Table 3).

**TABLE 3:** Multivariable regression analysis with predictors of changes in right ventricular function after 6‐month therapy with sacubitril/valsartan in patients with chronic heart failure and left ventricular dysfunction

	multivariable regression analysis				
	Beta	Std. Err.	b	Std. Err.	*p*‐value
Changes in right ventricular free wall longitudinal strain (%)					
Male gender	0.0371	0.1170	0.0294	0.0927	0.7533
Age (years)	0.2845	0.1241	0.0093	0.0041	0.0284
LVEF (%)	−0.4607	0.1476	−0.0120	0.0038	0.0037
RAV max (ml)	−0.3192	0.1361	−0.0030	0.0013	0.0252
PAsP (mmHg)	−0.0038	0.1317	−0.0001	0.0037	0.9772
RV S’	0.7465	0.2692	0.1191	0.0429	0.0091
TAPSE (mm)	−0.5340	0.2703	−0.0513	0.0259	0.0566
FAC (%)	0.3605	0.2465	0.0165	0.0113	0.1531
Changes in LVEF (%)	0.1576	0.1307	0.1565	0.1298	0.2363
Changes in LVEDV (%)	−0.1724	0.1183	−0.2755	0.1890	0.1544
Changes in right ventricular 4‐chamber longitudinal strain (%)					
Male gender	0.0492	0.1178	0.0574	0.1376	0.6791
Age (years)	0.2376	0.1250	0.0115	0.0060	0.0661
LVEF (%)	−0.3702	0.1487	−0.0142	0.0057	0.0180
RAV max (ml)	−0.2709	0.1371	−0.0037	0.0019	0.0565
PAsP (mmHg)	−0.0221	0.1326	−0.0009	0.0056	0.8684
RV S’	0.9062	0.2712	0.2129	0.0637	0.0021
TAPSE (mm)	−0.5308	0.2723	−0.0750	0.0385	0.0598
FAC (%)	0.2272	0.2483	0.0153	0.0167	0.3669
Changes in LVEF (%)	0.0627	0.1316	0.0917	0.1925	0.6368
Changes in LVEDV (%)	−0.2498	0.1192	−0.5877	0.2804	0.0438

Abbreviations: FAC, fractional area change; LVEDV, Left Ventricular End‐Diastolic Volume; LVEF, left ventricular ejection fraction; PAsP, pulmonary arterial systolic pressure; RAV, right atrial volume; RV S’, right ventricular peak systolic velocity; TAPSE, tricuspid annular plane systolic excursion.

## 4 DISCUSSION

To the best of our knowledge this is the first study showing that, in a real‐life registry, echocardiography parameters may identify subjects treated with S/V whose RV function is expected to improve after 6 months of treatment. Baseline RV S’ and RAV values are independent predictors of RV improvement.

Among RV systolic function echocardiographic parameters, TAPSE, RV S’ and RV FAC are the most referenced and studied (Lang et al., 2015). Recently, we showed that, in a real‐world scenario, S/V may be associated with an improved RV function in terms of TAPSE and PAsP (Correale et al., 2020). However, TAPSE and RV S’ are angle dependent and only reflect the longitudinal function of the basal portion of the RV, not considering the contribution of the apical and outflow tract components (Zaidi et al., 2020). Furthermore, no significant modifications were observed concerning RV echo parameters in other studies (Bayard et al., 2019).

Our findings, however, are in line with previous studies. In patients with HFrEF, Masarone et al. (2020) demonstrated that S/V may improve RV‐pulmonary artery coupling; Landolfo et al. (2020) showed a PAsP improvement after S/V therapy and Yenerçağ et al. (2021) TAPSE, FAC and pulmonary artery stiffness improvement. In preclinical studies on rats with pulmonary hypertension, S/V reduced RV systolic pressure, RV hypertrophy and dilatation (Andersen et al., 2019). These effects may be secondary to pulmonary vascular changes, including reduced pulmonary vascular remodelling, as demonstrated by reduction of pulmonary vascular wall thickness in rats with pulmonary hypertension treated with S/V (Clements et al., 2019). S/V may also prevent maladaptive RV remodelling in a pressure overload rat model via amelioration of RV pressure rise and hypertrophy (Sharifi Kia et al., 2020). Therapy with S/V plus bosentan proved significantly superior beneficial effect to S/V or bosentan alone on vascular remodelling and severity of experimental PH (Chaumais et al., 2021).

Pulmonary hypertension due to left heart disease (PH‐LHD) frequently complicates HFrEF; S/V may increase levels of natriuretic peptides. The resulting action on natriuresis, diuresis and vasodilation may play an important role in the reduction of pulmonary pressures, thus affecting RV function.

Our results are even more interesting, because there is evidence that RV recovery in patients with HFrEF is associated with improved survival (Dini et al., 2016). However, data on RV positive remodelling after standard therapy for HFrEF are inconsistent. Tatli et al. found that carvedilol treatment for 4 months resulted in a significant improvement of RVEF in 74 patients with systolic HF (Tatli et al., 2008). On the other hand, in the Beta‐Blocker Evaluation of Survival Trial (BEST) on outpatients with chronic advanced HFrEF receiving renin‐angiotensin inhibition, digoxin and diuretics, with RVEF <20% in those receiving additional therapy with bucindolol, RVEF <20% had a significant independent association with increased risk of mortality (Desai et al., 2013).

No data, however, are available on possible predictors of RV improvement after S/V therapy with echocardiography and tissue Doppler imaging. In stable patients with LV systolic dysfunction, RV S’ may add prognostic information over RVEF and TAPSE and the determination of RV S’, in addition to RVEF, could improve risk stratification (de Groote et al., 2012).

In patients with chronic HFrEF, RAVI is a determinant of right‐sided systolic dysfunction (Sallach et al., 2009) and it provides independent risk prediction of long‐term adverse events (Darahim, 2014). RA enlargement is also a powerful and reproducible independent predictor of long‐term mortality in patients with HFrEF in sinus rhythm receiving cardiac resynchronization therapy (Altes et al., 2019).

The assessment of RV function at TDI and RAV may be therefore useful in identifying subjects potentially benefitting from S/V therapy in terms of RV function improvement, beyond effects on LV function. However, such preliminary data from a monocentric small population warrant further confirmation in larger cohorts, longer follow‐up periods and randomized studies.

## 5 CONCLUSIONS

In a real‐world scenario, S/V therapy was associated with an improved RV function assessed by tissue Doppler imaging modalities (speckle‐tracking strain) in HFrEF. Baseline RV S’ and right atrial volumes values are independent predictors of RV function improvement after 6‐month therapy with S/V.

### 5.1 Limitations

Main limitations of the study are represented by the small number of patients enrolled, the monocentric and observational nature of the study; these preliminary results need to be confirmed in a properly powered multicentric study.

## CONFLICT OF INTEREST

The authors have no conflict of interest to disclose.

## DATA AVAILABILITY STATEMENT

Data are available on request.
